# Supplementary material for: Tuning lower dimensional superconductivity with hybridization at a superconducting-semiconducting interface
Source: Nat Commun. 2022 Aug 1;13:4452. doi: 10.1038/s41467-022-31948-3 (PMC9343457; doi:10.1038/s41467-022-31948-3)
Supplement: Supplementary file 1 — Supplementary Information [file 41467_2022_31948_MOESM1_ESM.docx]

Supplementary Information for

**Tuning lower dimensional superconductivity with hybridization at a superconducting-semiconducting interface**

Anand Kamlapure^1^, Manuel Simonato^1^, Emil Sierda^1^, Manuel Steinbrecher^1^, Umut Kamber^1^, Elze J. Knol^1^, Peter Krogstrup^2^, Mikhail I. Katsnelson^1^, Malte Rösner^1,*^, Alexander Ako Khajetoorians^1,*^

*^1^ Institute for Molecules and Materials, Radboud University, 6525 AJ Nijmegen, the Netherlands*

*^2^ Center for Quantum Devices, Niels Bohr Institute, University of Copenhagen, 2100 Copenhagen, Denmark*

*corresponding authors: [a.khajetoorians@science.ru.nl](mailto:a.khajetoorians@science.ru.nl), m.roesner@science.ru.nl

**Contents**

[Supplementary note 1: Simulation of the moiré pattern 4](#_Toc105582501)

[Supplementary note 2: Analysis of the Fermi surface and band structure within *ab Initio* calculations- 4](#_Toc105582502)

[Supplementary note 3: Superconducting spectral function for anisotropic dispersions 5](#_Toc105582503)

[Supplementary Fig. 1. Spectroscopy on Pb islands and BP within Pb vacancy island for closed film. 7](#_Toc105582504)

[Supplementary Fig. 2. Overview of the growth of Pb(111) films on BP(001) and the distribution of film thickness. 8](#_Toc105582505)

[Supplementary Fig. 3. Spectroscopy across the Pb film and BP. 9](#_Toc105582506)

[Supplementary Fig. 4. Simulation of the moiré pattern. 10](#_Toc105582507)

[Supplementary Fig. 5. Vortex imaging at different bias voltage. 11](#_Toc105582508)

[Supplementary Fig. 6. Vortex imaging of a 30ML Pb film. 12](#_Toc105582509)

[Supplementary Fig. 7. Measurement of the upper critical field and the coherence length. 13](#_Toc105582510)

[Supplementary Fig. 8. Lattice structure of the utilized supercell. 14](#_Toc105582511)

[Supplementary Fig. 9. Spectroscopy along a line measured on various Pb films and thicknesses. 15](#_Toc105582512)

[Supplementary Fig. 10. Comparison of spectroscopy on Pb islands and closed Pb films. 16](#_Toc105582513)

[Supplementary Fig. 11. Evolution of the LDOS with varying film thickness. 17](#_Toc105582514)

[Supplementary Fig. 12. Modeling superconducting gaps using a two-band hybrid model. 18](#_Toc105582515)

[Supplementary Fig. 13. Thickness-dependence of the superconducting gap. 19](#_Toc105582516)

[Supplementary Fig. 14. Bandgap measurement of pristine BP samples. 20](#_Toc105582517)

[Supplementary Table S1: Parameters used to fit the spectra in Supplementary Fig. 12. 21](#_Toc105582518)

[Supplementary references 22](#_Toc105582519)

# Simulation of the moiré pattern

To simulate the experimentally observed moiré patterns, we first generated a real space image of a single layer of Pb(111) and the topmost layer of a BP(001) lattice ^1, 2^. We used the following function to generate a hexagonal lattice for Pb(111).

|  | $f_{Pb}=\frac{1}{9}+\frac{8}{9}\left[ \cos\left( \frac{1}{2}\boldsymbol{k}_{1}\boldsymbol{r} \right)\cos\left( \frac{1}{2}\boldsymbol{k}_{2}\boldsymbol{r} \right)\cos\left( \frac{1}{2}\boldsymbol{k}_{3}\boldsymbol{r} \right) \right].$ | (1) |
| --- | --- | --- |

Here, $\boldsymbol{k}_{n}\boldsymbol{r}=xk\cos\left( \theta+\frac{n\pi}{3} \right)+yk\sin\left( \theta+\frac{n\pi}{3} \right)$, where n = 1-3, $\theta$ is the rotation angle for the lattice ($\theta=0$ in our case) and $k=\frac{2\pi}{\left( \sqrt{3}/2 \right)a_{Pb}}$ , with $a_{Pb}=$ lattice constant for Pb = 3.5 Å.

For the BP lattice we used the following function which generates two offset rectangular sublattices mimicking the top surface of the BP crystal:

|  | $f_{BP}=p_{1}+p_{2}\left[ \cos\left( xk_{b} \right)+\cos\left( yk_{a} \right) \right]^{m}+p_{3}\left[ \cos\left( xk_{b}+d \right)+\cos\left( yk_{a}+a/2 \right) \right]^{n}.$ | (2) |
| --- | --- | --- |

Here, $k_{a}=\frac{2\pi}{a}$,$k_{b}=\frac{2\pi}{b}$, with $a$ = 3.313 Å, *b* = 4.374 Å, *d* = sublattice offset $=$ 1.48 Å, $p_{1}=$ constant offset, $p_{2,3}=$ coefficient for the two offset lattices and $m,n=$ power indices are for the two sublattices that are needed to generate a corrugation that is comparable to experimental STM topograph. The two lattices were generated with a size of 40 nm x 40 nm on a grid with 2000 x 2000 pixels. Supplementary Fig. 4(a,b) shows the generated Pb(111) and BP(001) lattices.

The resulting moiré pattern was then calculated as a convolution of both lattices,

|  | $M=f_{Pb}\times f_{BP}.$ | (3) |
| --- | --- | --- |

Before comparing resulting pattern with experiments, we smoothed the generated image using a two-dimensional Gaussian filter. The resulting moiré image is shown in Supplementary Fig. 4c. We could capture all the essential features such as the moiré supercell size, vertically elongated shapes, etc., by adding ~1% of strain in the Pb lattice along the zigzag direction of the BP lattice, using the following parameters: $p_{2}=$ 0.12, $p_{3}=$ 0.2, $m=$ 1 and $n=$ 2.

# Analysis of the Fermi surface and band structure within *ab Initio* calculations-

To analyze the Fermi surfaces and band structures of the heterostructures we unfold the spectral functions of the supercells to the Brillouin zone of the primitive triangular unit cell of the Pb films using the approach from Ref. ^3^ according to

|  | $A(k,\omega)=\sum_{m} P_{Km}(k)\delta(E_{m}-\omega) \text{with}\text{ }P_{Km}(k)=\sum_{n} \left\vert\left\langle Km\vert kn \right\rangle\right\vert^{2}.$ | (4) |
| --- | --- | --- |

Here,$|\left. kn \right\rangle$ and $|\left. Km \right\rangle$ are Kohn-Sham states of the primitive and super cells, respectively. For the unfolded Fermi surfaces shown in the main text (Fig. 3), we plot $A(k,\omega\approx E_{f})$ as a colormap for the whole primitive first Brillouin zone. In Fig. 4(a,b) of the main text, we additionally show the resulting band structures, where the dot size corresponds to the value of $A(k,\omega)$ at the given $k$-point and energy$\omega$. To analyze the resulting spectral function in comparison to the experimental STS spectra, we finally define the quasi-local spectral function as

|  | $\overset{\sim}{\rho}(\omega)=\sum_{k} g(k)A(k,\omega) \text{with} g(k)=\frac{1}{\sigma_{k}\sqrt{2\pi}}exp\left( -\frac{1}{2}\frac{k^{2}}{\sigma_{k}^{2}} \right)$ | (5) |
| --- | --- | --- |

with$\sigma_{k}$ defining the range of significant $k$ points around $\Gamma.$

In Fig. 4(a,b) of the main text, we show the unfolded band structures and quasi-local spectral functions for 5 and 6 Pb monolayers. In red we present the free-standing data and in blue the unfolded spectra including the effects of the BP substrate. In the case of 5 Pb monolayer, we can clearly identify the quantum well state as a rather flat band around $E_{F}$ at$\Gamma$, which leaves a clear footprint in $\overset{\sim}{\rho}(\omega)$ (for$\sigma_{k}=0.01$). Towards $M$ and $K$ this state starts to disperse by first increasing and afterwards decreasing in energy, whereby it crosses the Fermi level. Due to the finite strain of 1% in $x$-direction a slight asymmetry is imprinted. Upon including the BP substrate, this former well-defined quantum well state is heavily deformed and so are those states, which cross the Fermi level. In detail, by comparing the free-standing (red) band structure to the unfolded one of the heterostructure (blue), we see that a variety of new BP states appear, e.g. between $0$ and $0.5 eV$ between $M$ and $\Gamma$ or between $0.5$ and $1.25 eV$ around $\Gamma$. In the latter range we see three BP bands, which results from using three BP layer as the substrate. Especially the BP and Pb states around the Fermi level hybridize strongly, which significantly broadens the corresponding feature in$\overset{\sim}{\rho}(\omega)$. Furthermore, the additional BP states at slightly higher energies around $\Gamma$ gain significant weight in$\overset{\sim}{\rho}(\omega)$. For the 6 Pb layer case, we can observe a similar behavior, but here the quantum well state is shifted downward in energy (to around $-0.75 eV$ at$\Gamma$) while another one appeared around $+1.0 eV$ at$\Gamma$.

Our DFT calculations for the deformation of the Fermi surface away from $\Gamma$ are most reliable, the details around $\Gamma$ strongly depend on computational approximations. This is due to the peculiar characteristics of the quantum well states in Pb. As visible from the band structure calculations of pristine Pb films shown as red solid lines in Fig. 4, there are rather flat bands at $\Gamma$, which are close to *E*_F_ for odd layers and shifted away from *E*_F_ (and split) for even layers. These flat bands together with the tunneling sensitivity to states around $\Gamma$ form the well-known QWS seen as well-defined peaks in STS with a bi-layer oscillation. The exact energetic positions of these QWS however strongly depend on the exact doping levels, the intrinsic strain, as well as on the chosen approximations within the DFT calculations. For the case of the 5 ML Pb, as the depicted in Fig. 3a of the main text, the QWS around Gamma is very close to *E*_F_ which yields together with the broadening a round feature in the Fermi surface map. As this QWS state strongly hybridizes with the BP conduction states in our calculations and as the depicted Fermi surfaces are taking all states in an energy windows of $\pm33$ meV around the Fermi level into account, the Fermi surface around $\Gamma$ is reshaped as shown in Fig. 3b in the main text, which yields a broadened QWS fingerprint in the calculated STS spectrum close to *E*_F_. Since this feature is not prominently visible in the experimental STS data for 5 ML Pb and since it is highly dependent on the exact initial positions of the Pb QWS as well as on the underlying BP band structure, we interpret this as a modeling artefact. Finally, we note that the modifications to the outer Fermi surface areas (away from $\Gamma$) in a heterostructure consisting of 6ML Pb on 3 ML BP (which doesn’t suffer from details of states around $\Gamma$ and close to *E*_F_) are very similar to those in 5 ML Pb on 3 ML BP: Considering negligible variations in the superconducting properties between even and odd layers for a given sample growth, we argue that the BP-Pb hybridization with Pb states away from $\Gamma$ matter most for the modifications to the SC properties of Pb.

# Superconducting spectral function for anisotropic dispersions

Given the dispersion $E_{k}$ the spectral function is defined by

|  | $\rho(\omega)=\frac{1}{4\pi^{2}}\int d\mathbf{k}\delta\left( \omega-E_{k} \right)=\frac{1}{4\pi^{2}}\int_{\Gamma} d\gamma\frac{1}{\vert\nabla E_{k}\vert}$ | (6) |
| --- | --- | --- |

with$\Gamma$ being the set $k_{\Gamma}$ of $k$-points satisfying$\omega=E_{k}$.

For conventional superconductors with $E(k,\theta)=\sqrt{\zeta^{2}(k,\theta)+\Delta^{2}(k,\theta)}$ (in two dimensions and in polar coordinates) the range in which the pairing amplitude $\Delta$ significantly differs from zero is generally limited in $k$-space to a narrow stripe around the Fermi surface of width $|\zeta_{k}|<\omega_{D}$with $\omega_{D}$ being the Debye frequency. We can thus approximate $\Delta(k,\theta)\simeq\Delta(k_{F},\theta)\equiv\Delta(\theta)$.

We proceed with a generic anisotropic quadratic dispersion using the effective masses $m_{x}^{*}>m_{y}^{*}$ and utilizing polar coordinates

|  | $\zeta_{k}=\frac{1}{2m_{x}^{*}}k_{x}^{2}+\frac{1}{2m_{y}^{*}}k_{y}^{2}-\mu=\frac{1}{2m_{x}^{*}}k^{2}\underset{=G\left( \theta\right)}{\underbrace{\left[ 1+\epsilon\sin^{2}\left( \theta\right) \right]}}-\mu.$ | (7) |
| --- | --- | --- |

Here, $\mu$ denotes the chemical potential and $\epsilon=\frac{m_{x}^{*}}{m_{y}^{*}}-1$. The function $G(\theta)$ characterizes the anisotropy in the dispersion which modulates the Fermi surface as a function of $\theta$according to the Fermi wavevector

|  | $k_{F}\left( \theta\right)=\sqrt{\frac{2m_{x}^{*}\mu}{G\left( \theta\right)}}=\frac{k_{0}}{\sqrt{G\left( \theta\right)}}$ | (8) |
| --- | --- | --- |

with $k_{0}$ being the Fermi wavevector of an isotropic dispersion with effective mass $m_{x}^{*}$. With this, the set $k_{\Gamma}$ that satisfies $E^{2}(k_{\Gamma},\theta)=\zeta^{2}(k_{\Gamma},\theta)+\Delta^{2}(\theta)=\omega^{2}$ is given by

|  | $k_{\Gamma}^{2}\left( \omega,\theta\right)=k_{F}^{2}\left( \theta\right)\pm\frac{2m_{x}^{*}}{F\left( \theta\right)}\sqrt{\omega^{2}-\Delta^{2}\left( \theta\right)}\approx k_{F}^{2}\left( \theta\right).$ | (9) |
| --- | --- | --- |

The latter approximation is justified since we are mostly interested in the energy range of $\omega\approx\Delta(\theta)$. With this we can approximate the denominator in Supplementary Eq. (6) $\nabla E_{k}$ as

|  | $\nabla E\left( k_{F}\left( \theta\right) \right)\approx\frac{\left\vert\zeta\left( k_{F}\left( \theta\right) \right) \right\vert}{\sqrt{\zeta^{2}\left( k_{F}\left( \theta\right) \right)+\Delta^{2}\left( \theta\right)}}\left\vert\nabla\zeta\left( k_{F}\left( \theta\right) \right) \right\vert=\frac{\sqrt{\omega^{2}-\Delta^{2}\left( \theta\right)}}{\left\vert\omega\right\vert}\left\vert\nabla\zeta\left( k_{F}\left( \theta\right) \right) \right\vert,$ | (10) |
| --- | --- | --- |

where we additionally assumed that the $\Delta(\theta)\nabla\Delta(\theta)$ term is negligibly small. With $\left| \nabla\zeta\left( k_{F}\left( \theta\right) \right) \right|\simeq\frac{1}{m_{x}^{*}}G(\theta)k_{F}(\theta)=\frac{1}{m_{x}^{*}}k_{0}\sqrt{G(\theta)}$ we finally obtain

|  | $\rho\left( \omega\right)=\rho_{0}\frac{1}{2\pi}\int_{0}^{2\pi} d\theta\underset{=w\left( \theta\right)}{\underbrace{\left[ \frac{1}{G\left( \theta\right)} \right]}}\frac{\left\vert\omega\right\vert}{\sqrt{\omega^{2}-\Delta^{2}\left( \theta\right)}} .$ | (11) |
| --- | --- | --- |

Here, $\rho_{0}=\frac{m_{x}^{*}}{2\pi}$ denotes the density of states of the isotropic parabolic dispersion relation in 2D and $w\left( \theta\right)$ is a weighting function as referred to in the main text. This expression for the superconducting spectral function is vastly reminiscent of the conventional one known for s-wave BCS superconductors, with the important difference that Supplementary Eq. (11) is also valid for anisotropic Fermi surfaces, which we here account for by introducing the function$G(\theta)$.

**Supplementary figures**


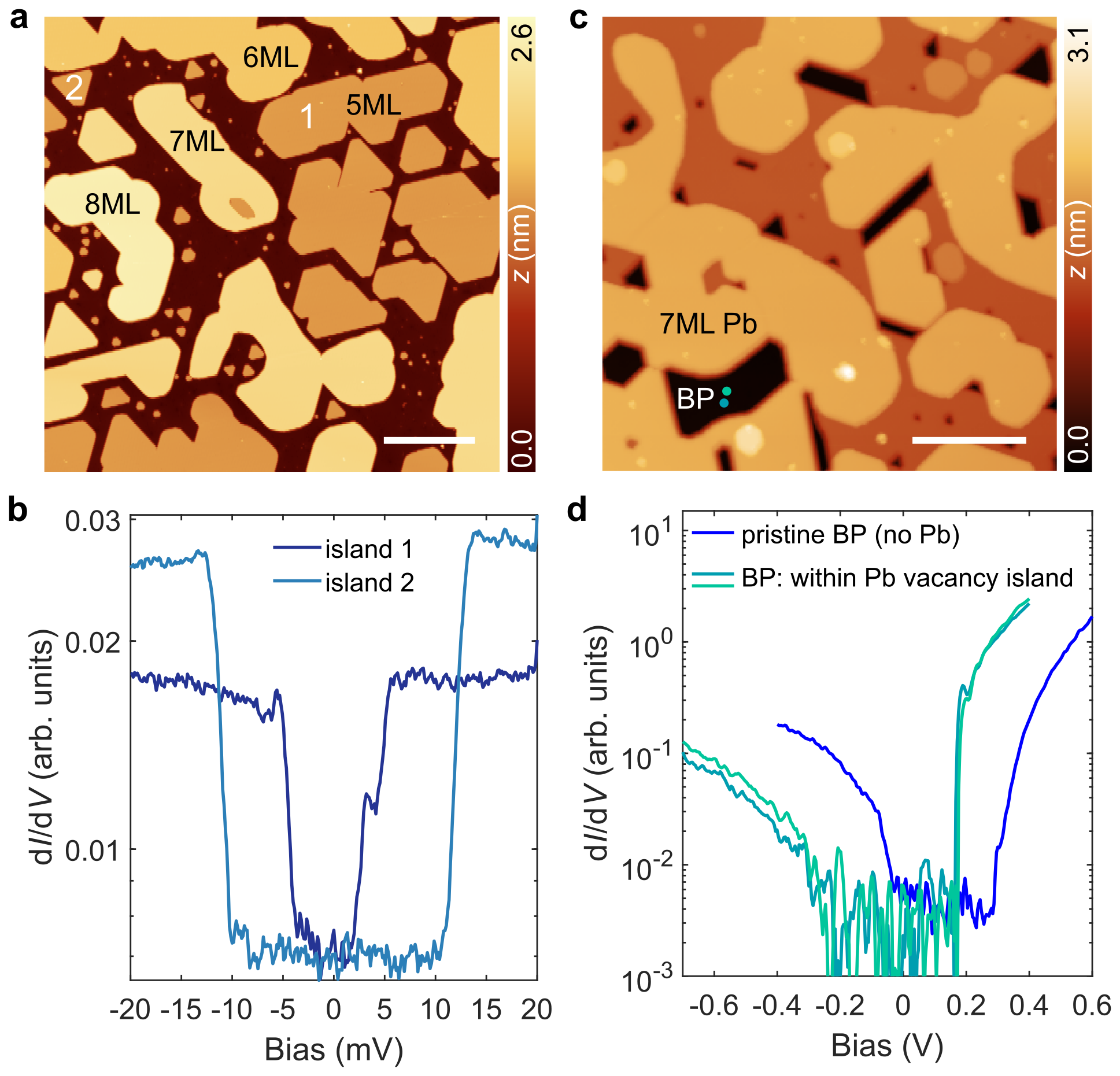


Supplementary Fig. 1. Spectroscopy on Pb islands and BP within Pb vacancy island for closed film. (**a**) Large scale STM image of Pb islands formed on BP when grown at room temperature. The minimum layer thickness observed for such room-temperature growth was 5 ML. (*V*_s_ = 600 mV, *I*_t_ = 20 pA, *T* = 1.3 K, scale bar = 100 nm). (**b**) d*I*/d*V* spectra measured on two islands each of thickness 5 ML. A hard gap close to the Fermi energy is a typical signature of Coulomb blockade, where the gap size increases as the island size is reduced. (*V*_stab_ = 320 mV, *I*_stab_ = 100 pA, *V*_mod_ = 200 µV, ∆z = -150 pm). (**c**) Constant current STM image showing typical Pb film with vacancy islands. (*V*_s_ = 600 mV, *I*_t_ = 10 pA, *T* = 1.3 K, scale bar = 50 nm). (**d**) d*I*/d*V* spectra measured on pristine BP before any Pb deposition (blue) and inside Pb vacancy island (green shades) measured at the locations marked in (**c**).


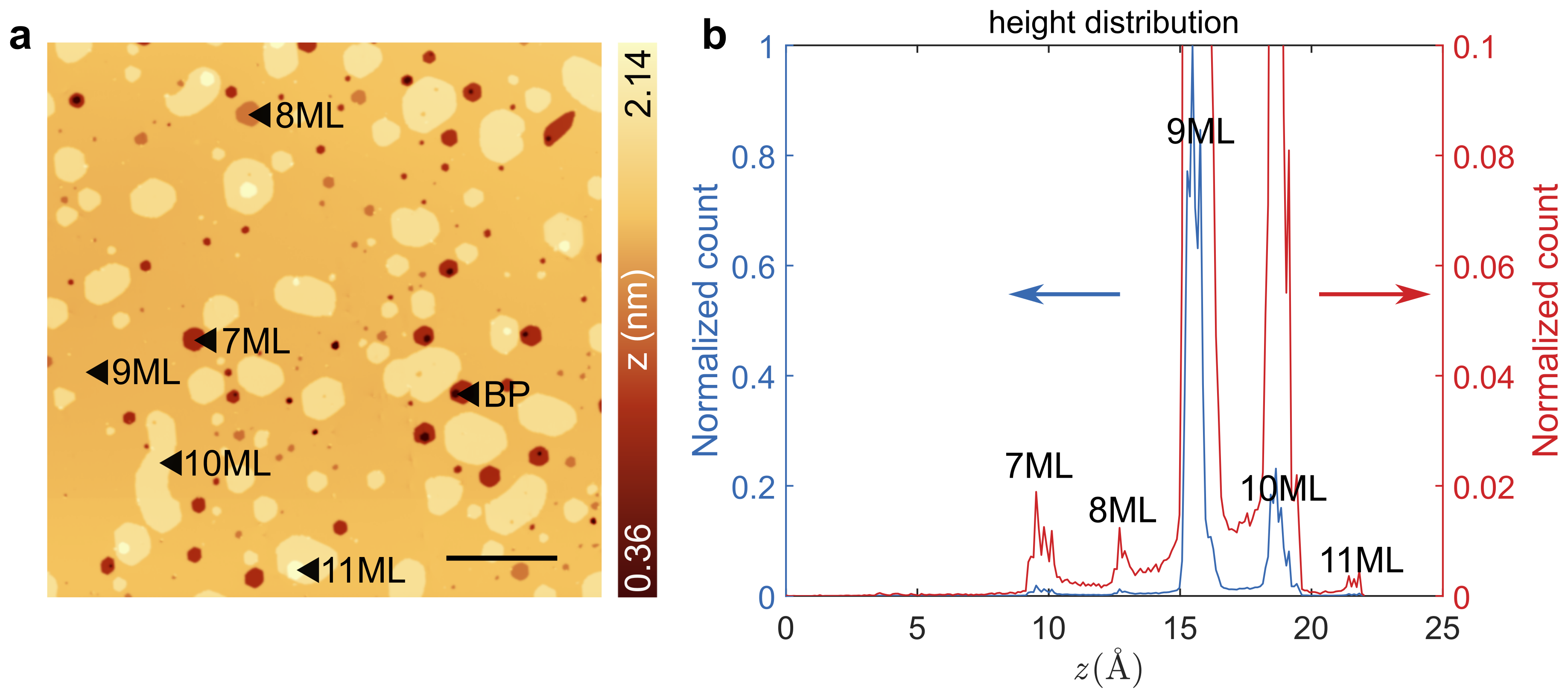


Supplementary Fig. 2. Overview of the growth of Pb(111) films on BP(001) and the distribution of film thickness. (**a**) Constant-current STM image of a typical growth of Pb on BP showing monolayer height variations. The image also shows holes penetrating all the way to BP. (*V*_s_ = 600 mV, *I*_t_ = 10 pA, *T* = 1.3 K, scale bar = 80 nm). (**b**) Histogram of the measured heights. Blue and red curves are the same, but plotted on two different y-scales to enhance smaller peaks. Peaks correspond to the different layer thicknesses. From the height distribution, it is clear that the dominant layer thickness for the given growth is 9 ML.


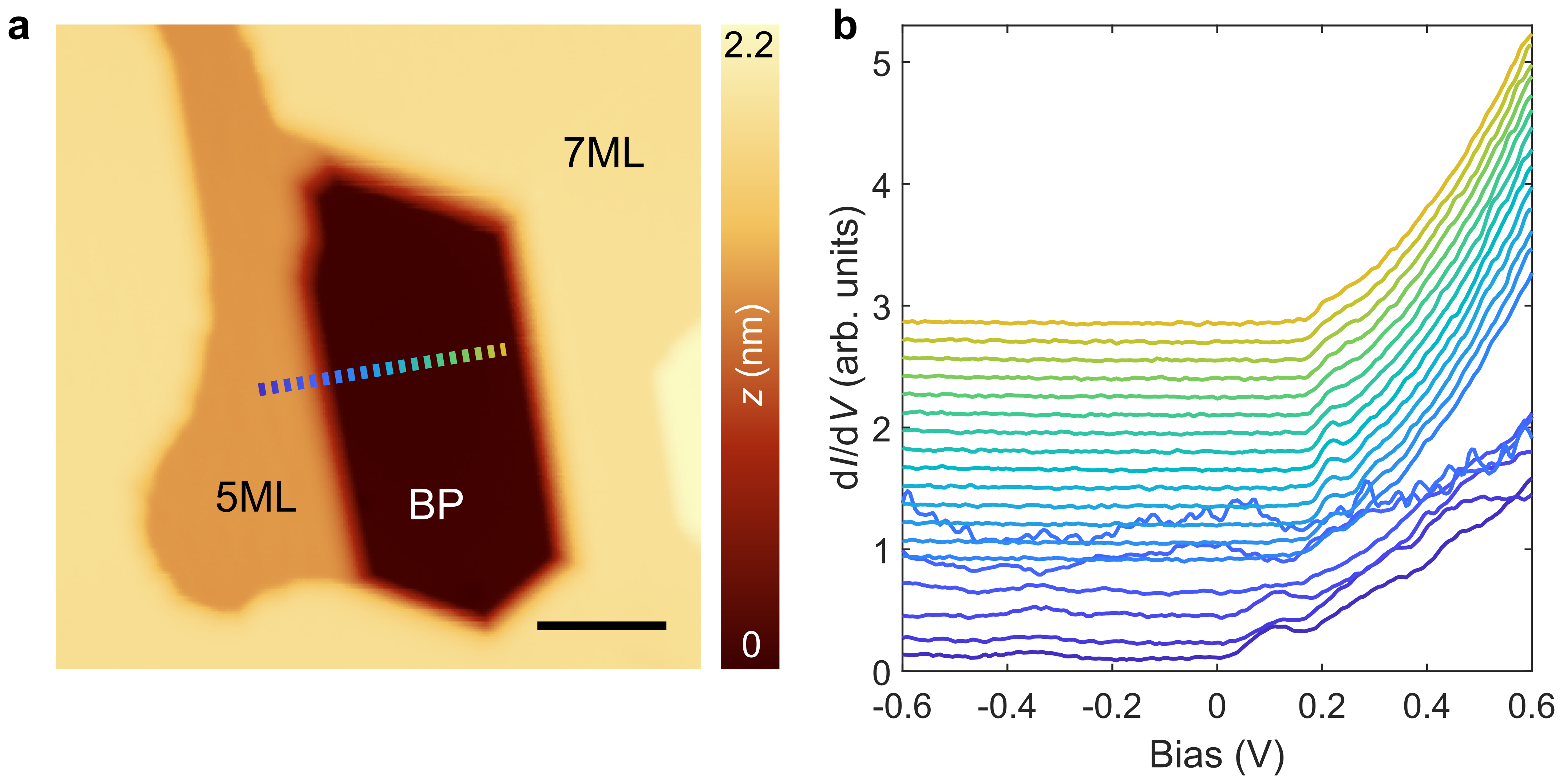


Supplementary Fig. 3. Spectroscopy across the Pb film and BP. (**a**) Constant-current STM image of a Pb film showing various film thicknesses together with a hole where the bare BP is exposed. (**b**) d*I*/d*V* spectra measured across the step from a 5 ML Pb film onto bare BP as shown in (a). Spectra are shifted vertically for clarity. Spectra on the 5 ML Pb film show typical spectral features corresponding to wide range spectroscopy (Fig. 4d). Spectra on BP show a bandgap around the Fermi energy with the conduction band onset at ~150 mV indicating no induced proximity effect in the substrate.


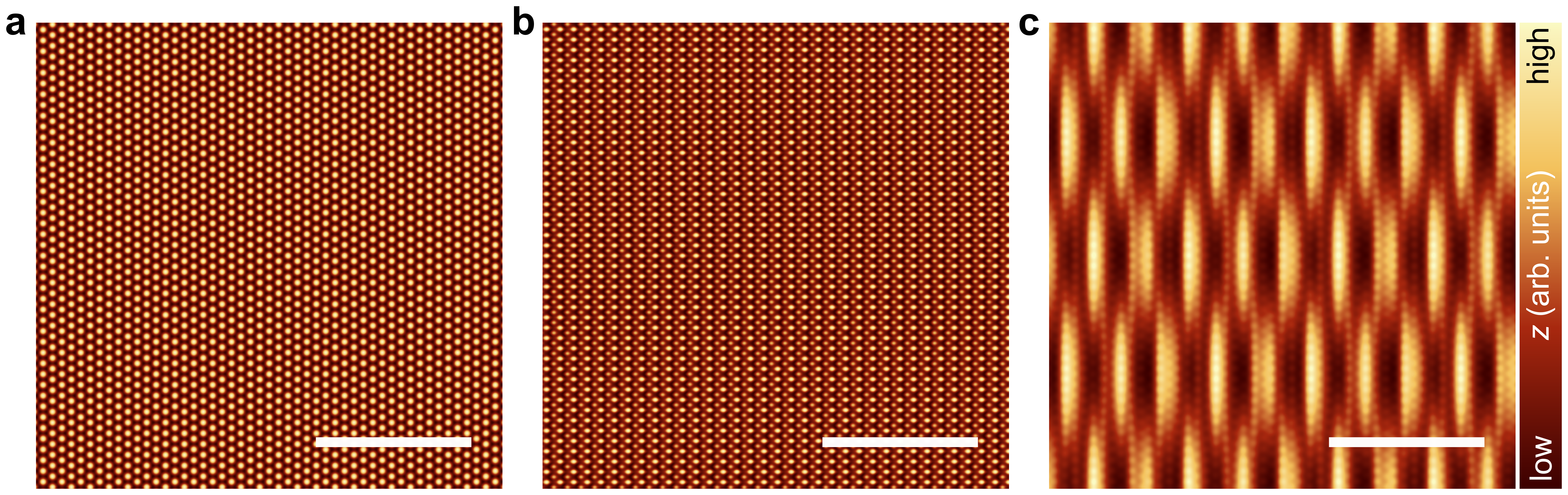


Supplementary Fig. 4. Simulation of the moiré pattern. (**a, b**) Simulated real space image of Pb and BP obtained using a combination of sinusoidal wave functions. (**c**) The Moiré pattern obtained by a convolution of images (a) and (b) followed by smoothing using a two-dimensional Gaussian filter. (Scale bar in each panel = 5 nm).


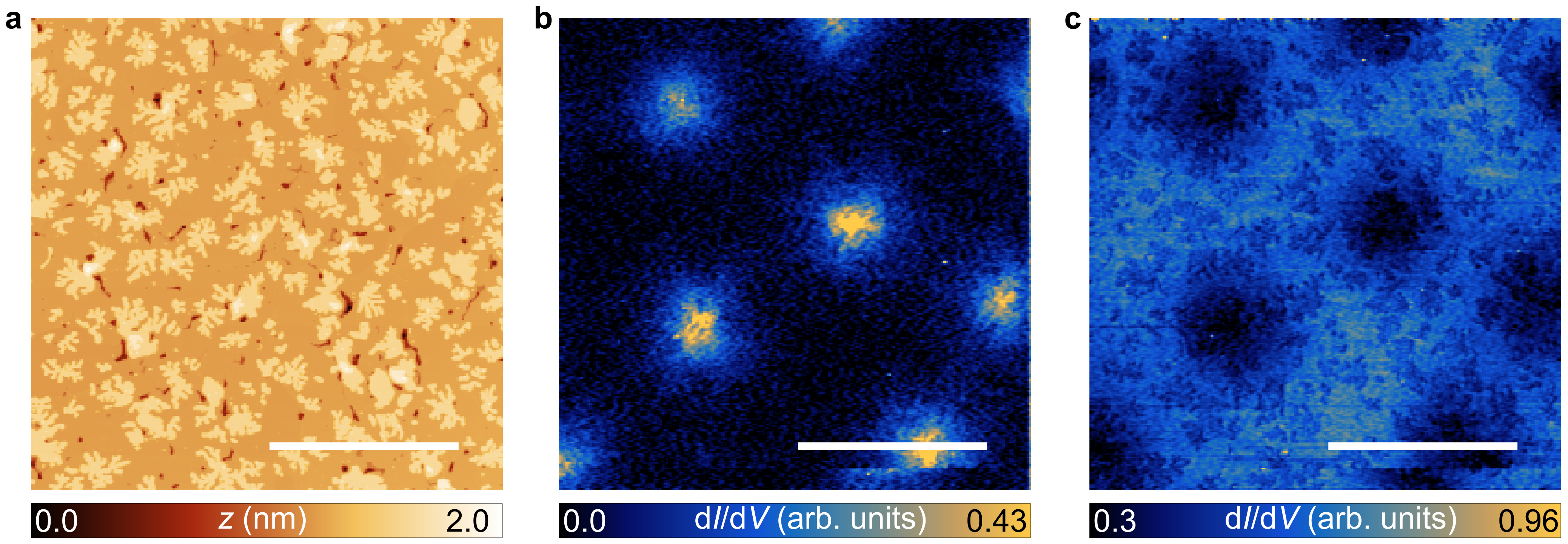


Supplementary Fig. 5. Vortex imaging at different bias voltage. (**a**) Constant-current STM image of the Pb film showing the topographic area studied for vortex imaging. Simultaneously measured d*I*/d*V* map at *V*_S_ = 0 V (**b**) and *V*_S_ = 1.3 mV (**c**) at *T* = 30 mK and $B_{\perp}$ = 50 mT, in the same area as Fig. 2b of the main manuscript. Imaging parameters *V*_stab_ = 10 mV, *I*_stab_ = 10 pA, *V*_mod_ = 100 uV, ∆*z* = - 80 pm (scale bar = 200 nm).


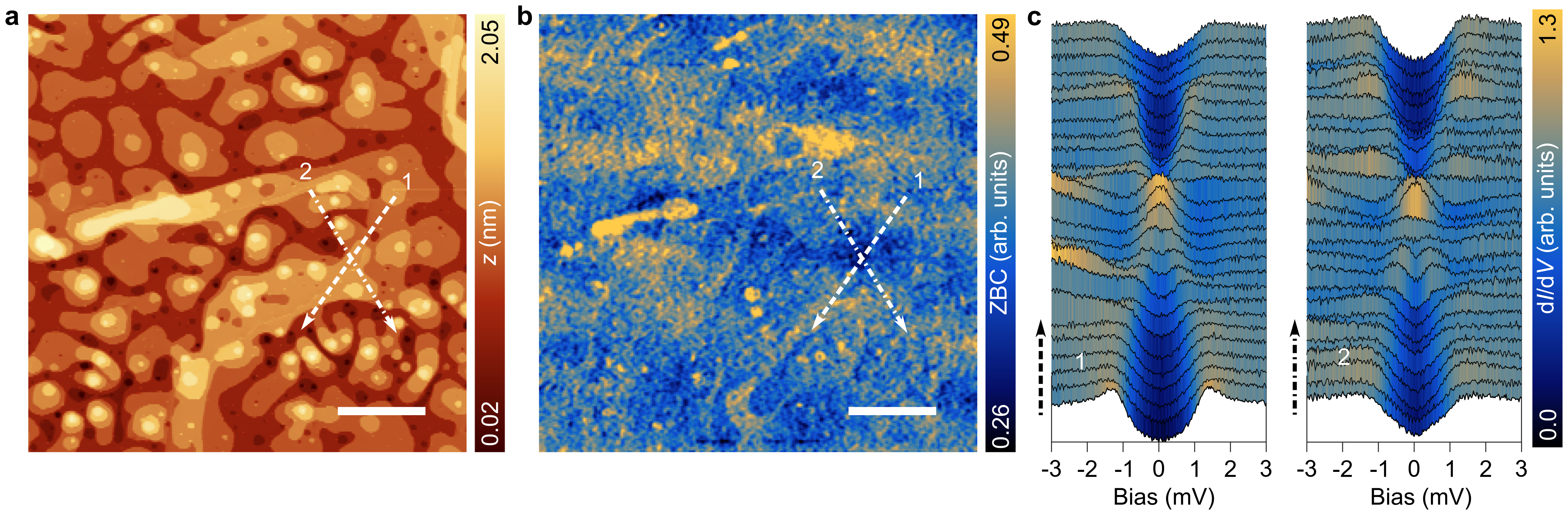


Supplementary Fig. 6. Vortex imaging of a 30ML Pb film. (**a**) Constant-current STM image of a thick Pb film (~30 ML) showing the topography of the area used for vortex imaging. (**b**) Zero bias conductance (ZBC) map at *T* = 30 mK and *H* = 50 mT showing a diffused vortex lattice. Imaging parameters for (a) and (b): *V*_stab_ = 10 mV, *I*_stab_ = 10 pA, *V*_mod_ = 200 µV, ∆z = - 80 pm (scale bar = 100 nm). (**c**) d*I*/d*V* spectra measured across a vortex in two directions (1,2) along the lines through the vortex in (b), each 183 nm in length. (*V*_stab_ = 5 mV, *I*_stab_ = 200 pA, *V*_mod_ = 50 µV).


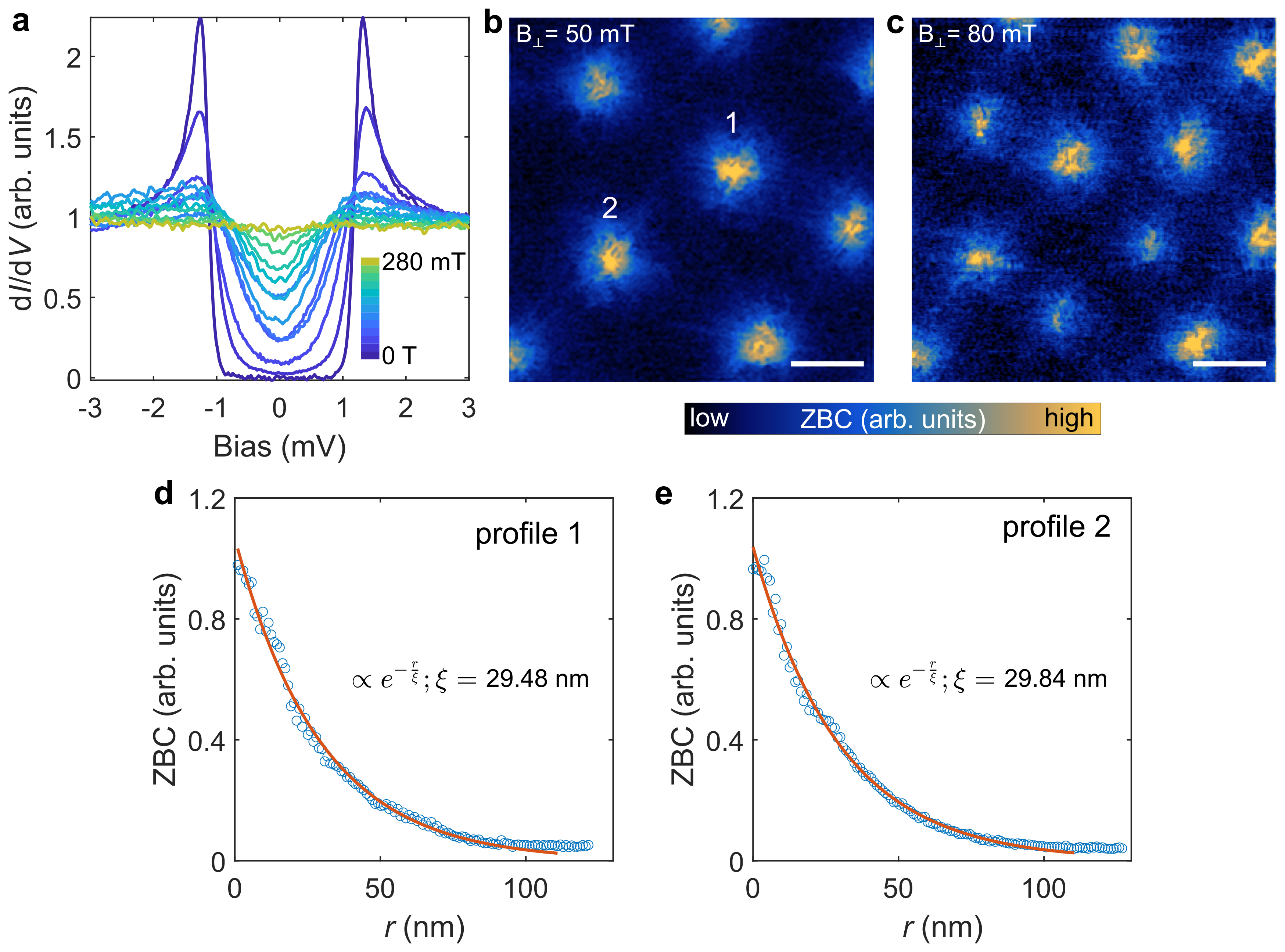


Supplementary Fig. 7. Measurement of the upper critical field and the coherence length. (**a**) d*I*/d*V* spectra measured as a function of out-of-plane magnetic field, measured away from vortices for as grown 7.3 ML Pb film. The superconducting gap vanishes at *H*_c2_ = 280 mT. We estimated the coherence length using $\xi=\sqrt{{\Phi_{0}}/{2\pi H_{c2}(0)}}=$ 34.3 nm, where $\Phi_{0}$ is the quantum of magnetic flux (*V*_stab_ = 5 mV, *I*_stab_ = 200 pA, *V*_mod_ = 50 µV, *T* = 35 mK). (**b, c**) Zero bias d*I*/d*V* map at B_⟂_ = 50 mT and B_⟂_ = 80 mT, showing an Abrikosov vortex lattice (panel (b) is same as Fig. 2b of the main manuscript). Imaging parameters for (b) and (c): *V*_stab_ = 10 mV, *I*_stab_ = 10 pA, *V*_mod_ = 200 uV, ∆z = - 80 pm, scale bar = 100 nm. (**d**, **e**) Radially averaged profiles across two vortices marked in (b). From an exponential fit of these profiles using a least squares method, the coherence length could be extracted: $\xi\sim$30 ±1.5 nm (95% confidence). This is consistent with the value determined using the upper critical field (*H*_c2_).


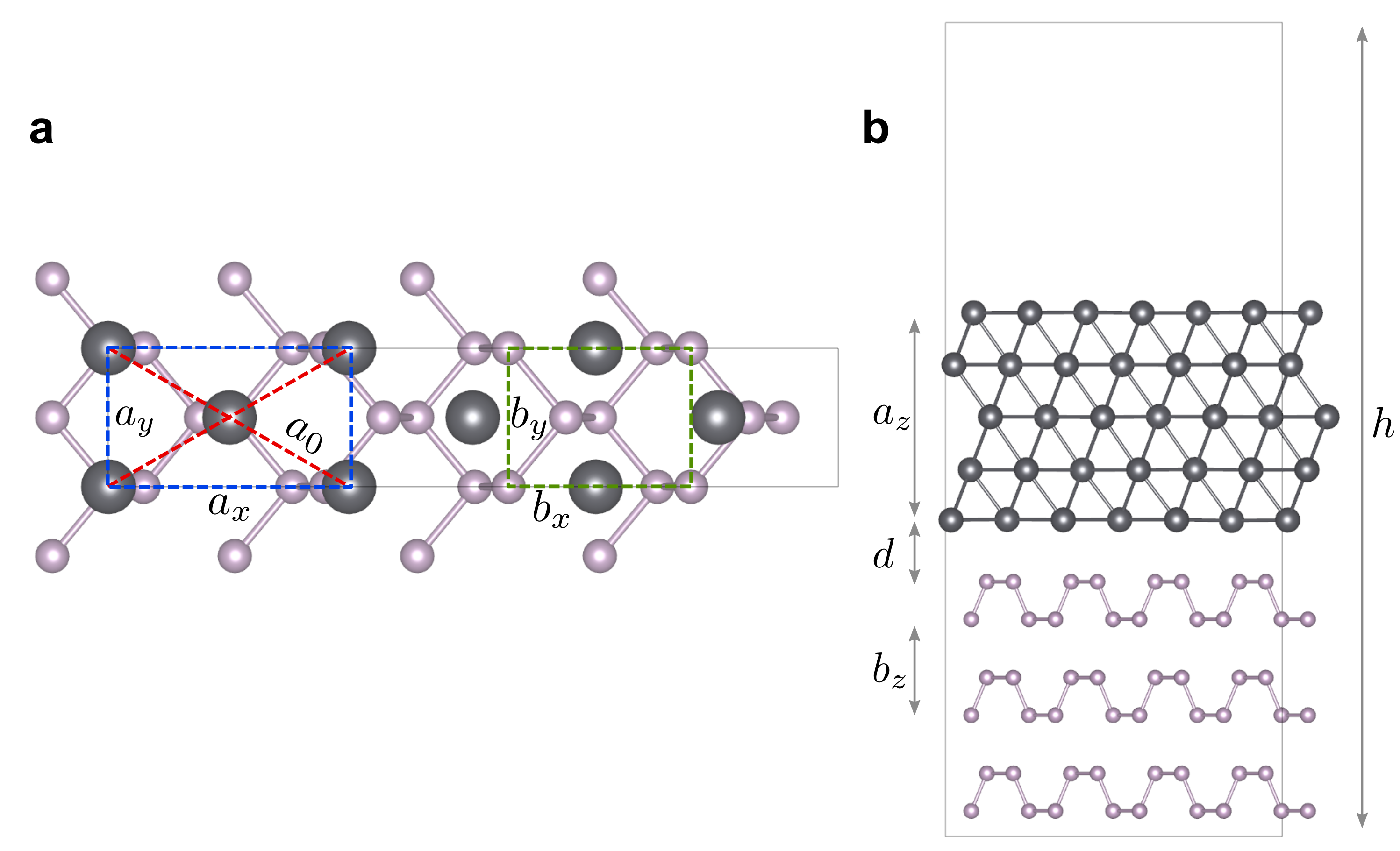


Supplementary Fig. 8. Lattice structure of the utilized supercell. (**a**) Top view of a supercell with a monolayer Pb on a monolayer of BP. Red, blue, and green dashed lines indicate the primitive (hexagonal) Pb(111), the rectangular Pb, and the primitive BP unit cells, respectively. (**b**) Side view of a supercell will 5 ML of Pb on top of 3 ML of BP.


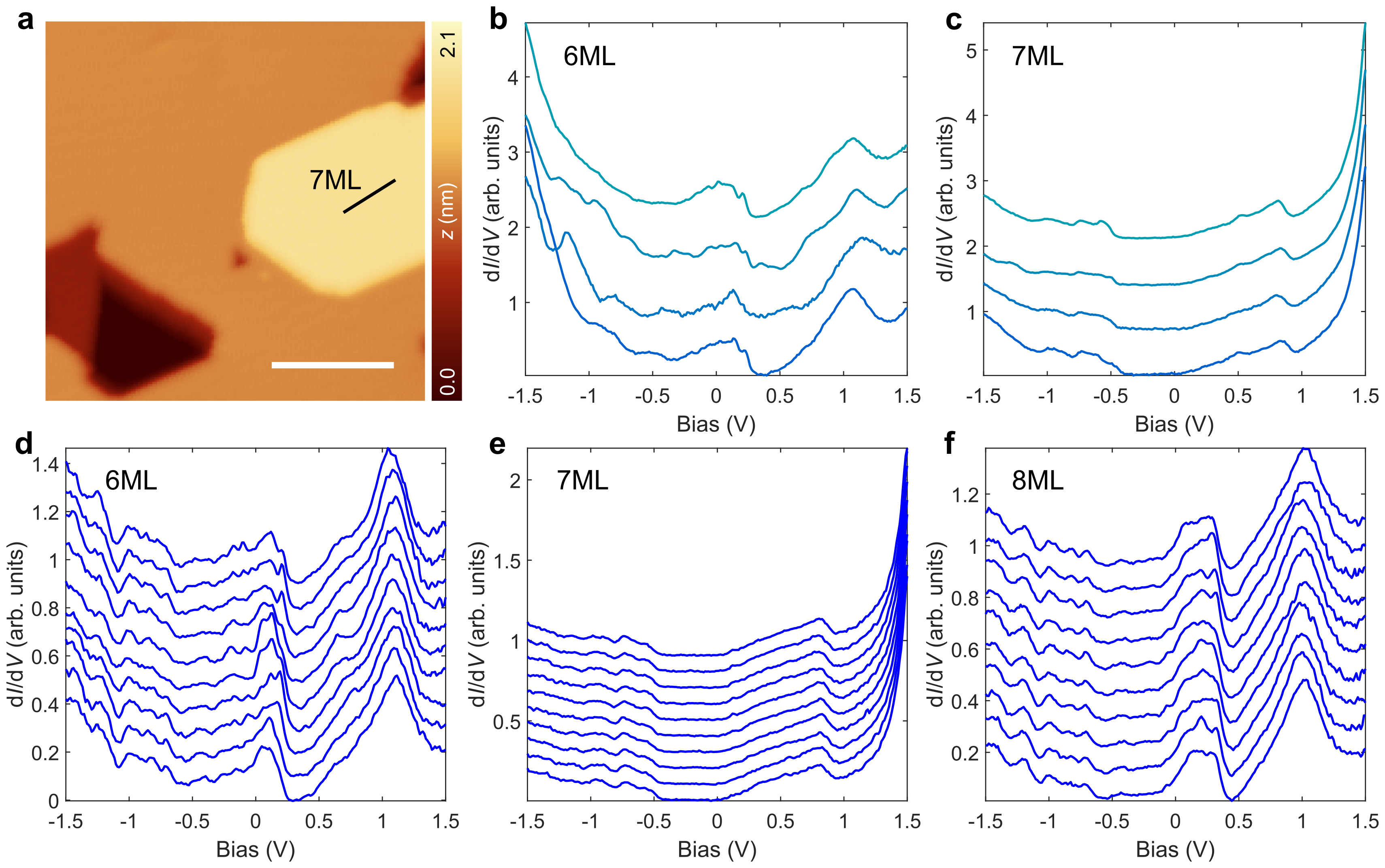


Supplementary Fig. 9. Spectroscopy along a line measured on various Pb films and thicknesses. (**a**) Constant current STM image of the Pb film showing typical line profile for spectroscopy measurements. (*V*_s_ = 600 mV, *I*_t_ = 10 pA, scale bar = 10 nm). (**b-c**) d*I*/d*V* spectra averaged over a line on 6 ML and 7 ML Pb films for different growths. (**d-f**) d*I*/d*V* spectra measured along a line for the same sample but various thicknesses as indicated in the panel. STS parameters for panels b, c: *V*_stab_ = 1.5 V, *I*_stab_ = 200 pA, *V*_mod_ = 20 mV, *T* = 1.3 K. STS parameters for panels d-f: *V*_stab_ = 1.5 V, *I*_stab_ = 200 pA, *V*_mod_ = 10 mV, *T* = 1.3 K All the spectra are shifted vertically for clarity and each color shade represents same sample growth.

**
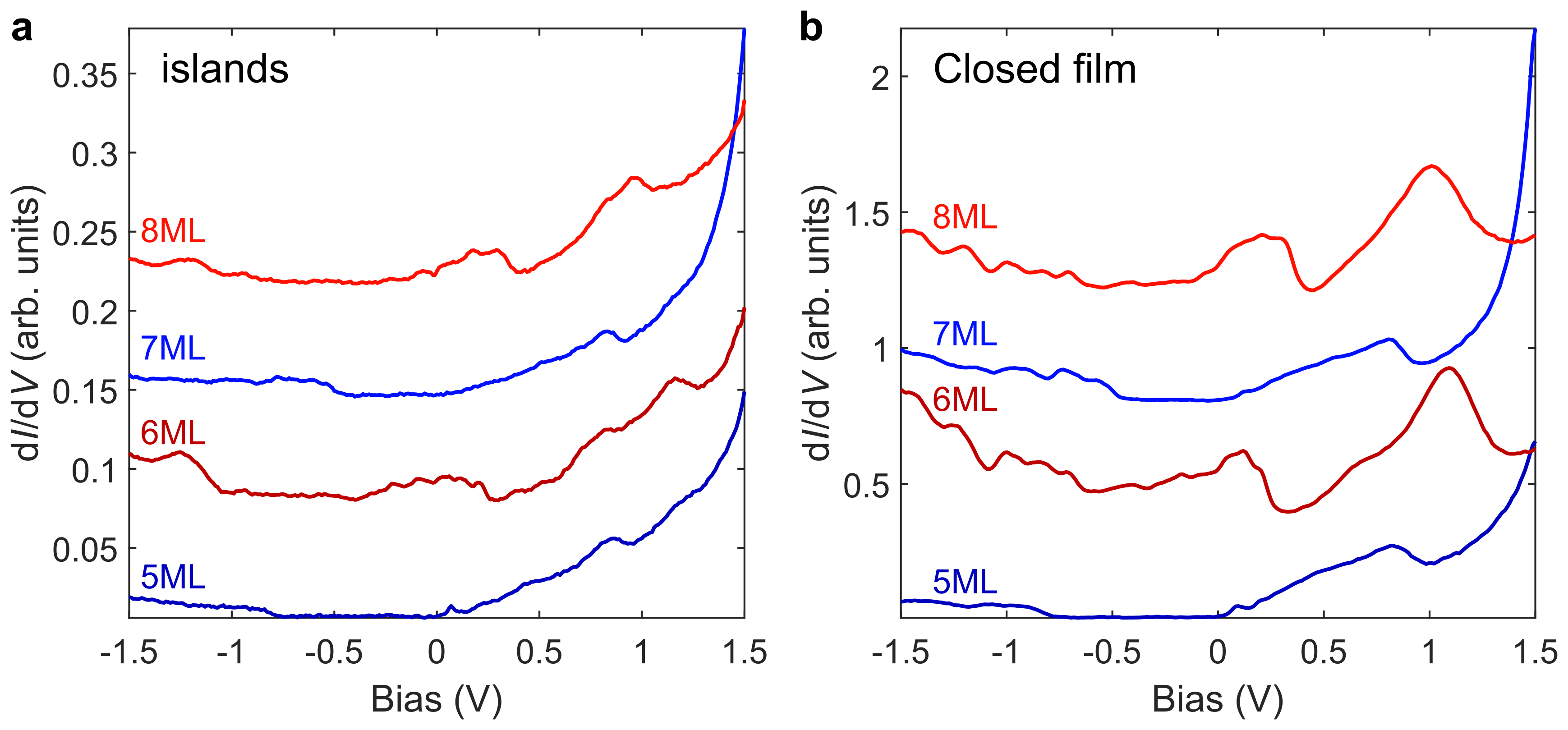
**

Supplementary Fig. 10. Comparison of spectroscopy on Pb islands and closed Pb films. Series of d*I*/d*V* spectra measured in a wide bias range for Pb islands (**a**) and Pb films (**b**) of thicknesses indicated on the left side of each curve. Even/odd films are plotted with same color shades (red/blue). Spectra are shifted vertically for clarity. STS parameters for panel a: *V*_stab_ = 1.5 V, *I*_stab_ = 200 pA, *V*_mod_ = 10 mV, *T* = 1.3 K. STS parameters for panel b: *V*_stab_ = 1.5 V, *I*_stab_ = 200 pA, *V*_mod_ = 20 mV, *T* = 1.3 K.


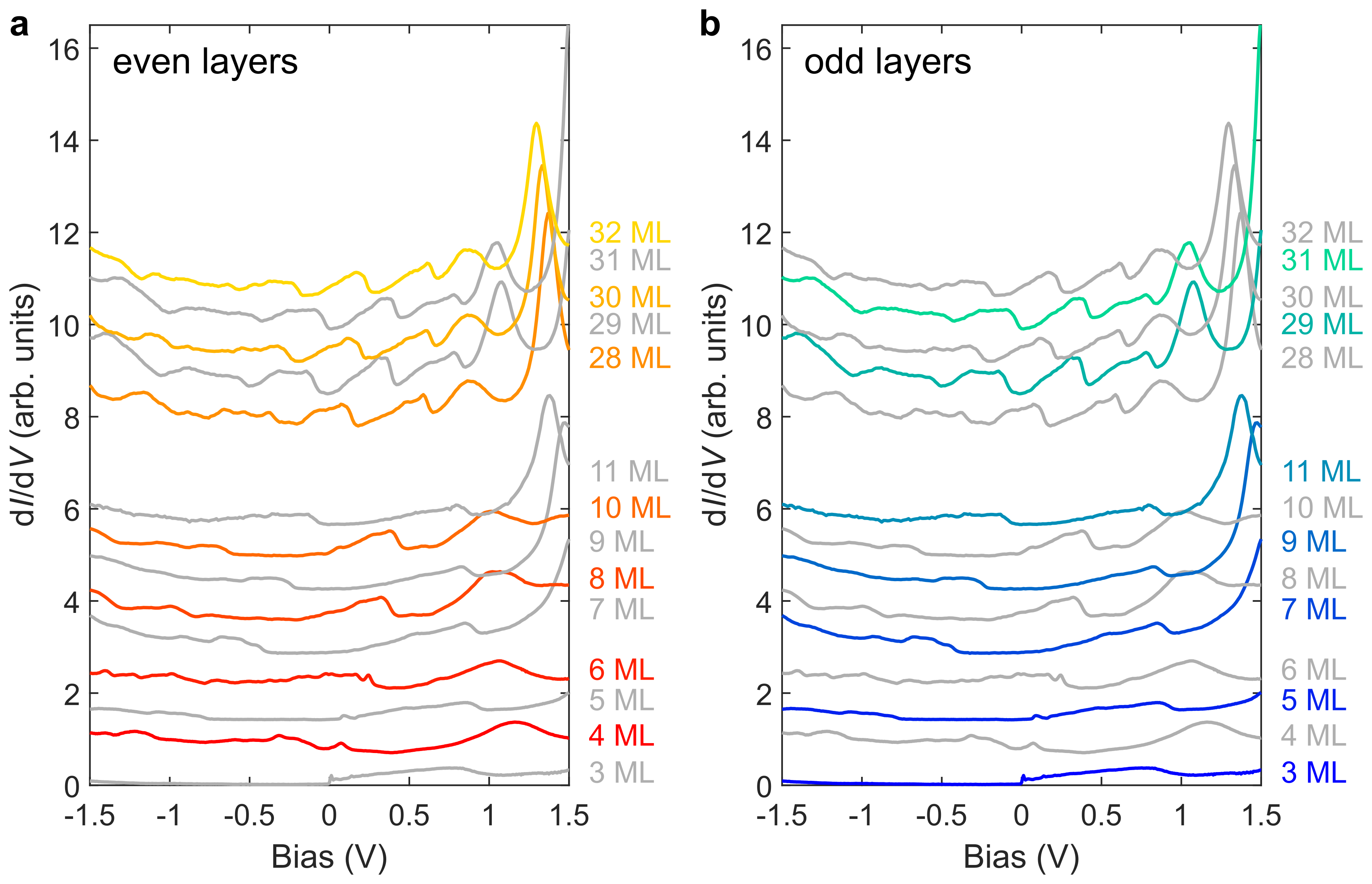


Supplementary Fig. 11. Evolution of the LDOS with varying film thickness. Series of d*I*/d*V* spectra measured in a wide bias range for Pb films of thicknesses indicated on the right of the panels. For clarity, same spectra are plotted in the two panels, where, even layer spectra are highlighted with red color shade in panel (a), while odd layer spectra are highlighted with blue color shade in panel (b). Opposite parity spectra in each panel are shown with gray color. All the spectra are shifted vertically. (stabilization parameters for 3-6, 28-32 ML: *V*_stab_ = 1.5 V, *I*_stab_ = 500 pA, *V*_mod_ = 5 mV; for 7-11 ML: *V*_stab_ = 1.5 V, *I*_stab_ = 200 pA, *V*_mod_ = 20 mV. Measurement temperature for 3-6 ML, 28-32 ML: *T* = 35 mK; measurement temperature for 7-11 ML: *T* = 1.3 K).


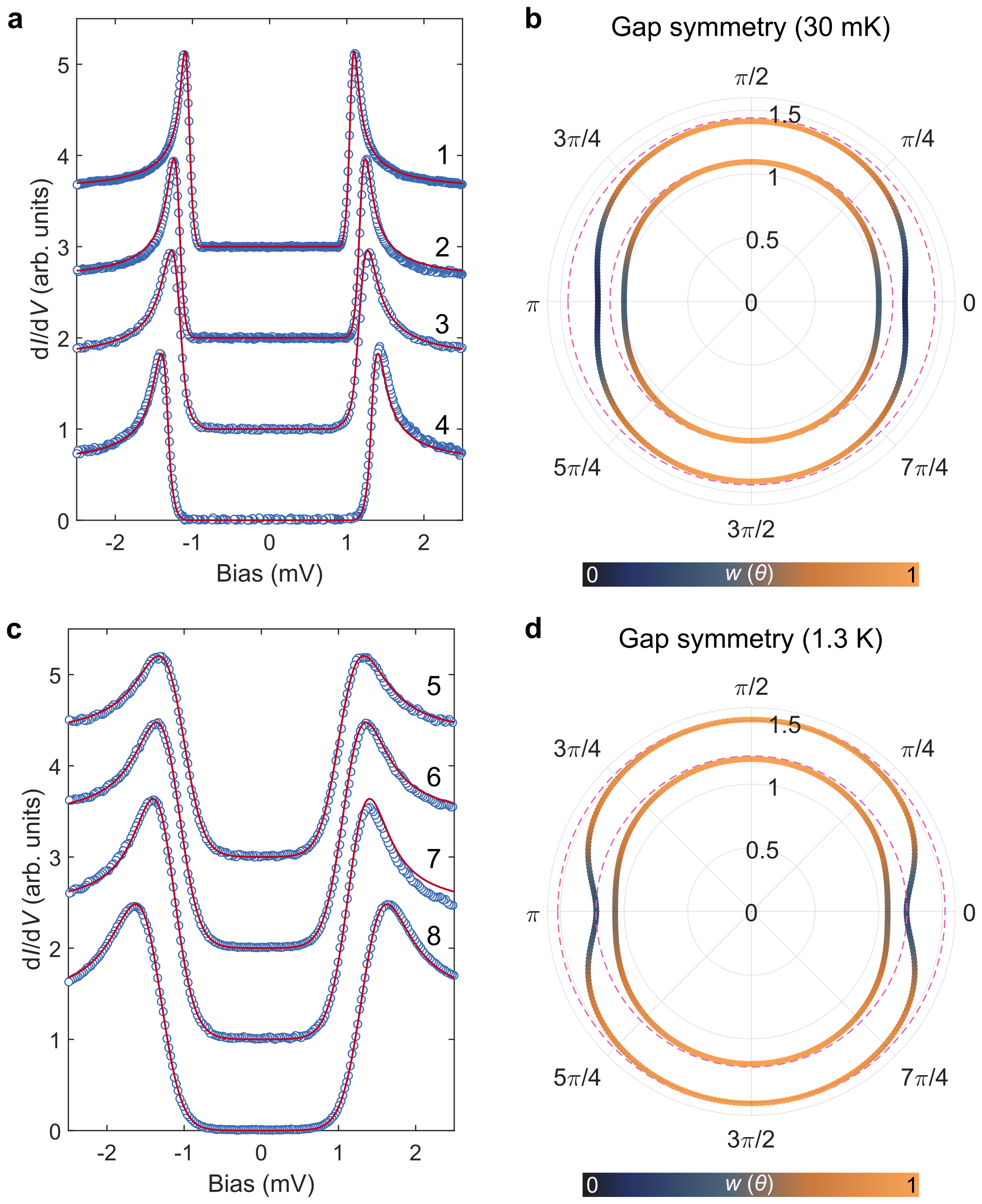


Supplementary Fig. 12. Modeling superconducting gaps using a two-band hybrid model. (**a,c**) d*I*/d*V* spectra (open blue circles) measured at *T* = 30 mK and *T =* 1.3 K respectively on different samples with similar layer thicknesses (6-7 ML). Spectra are shifted vertically for clarity. The solid red lines in (a) and (c) are fits to the corresponding spectra using an anisotropic gap and the weight function (*w* (*θ*)) as shown in the polar plots in (b) and (d) respectively. For clarity, (b) and (d) show the gap symmetry for large (outer curve) and small (inner curve) gaps only. Stabilization parameters: *V*_stab_ = 5 mV, *I*_stab_ = 200 pA, *V*_mod_ = 20-100 µV. The fitting parameters used in (a) and (c) are summarized in Supplementary Table 1.


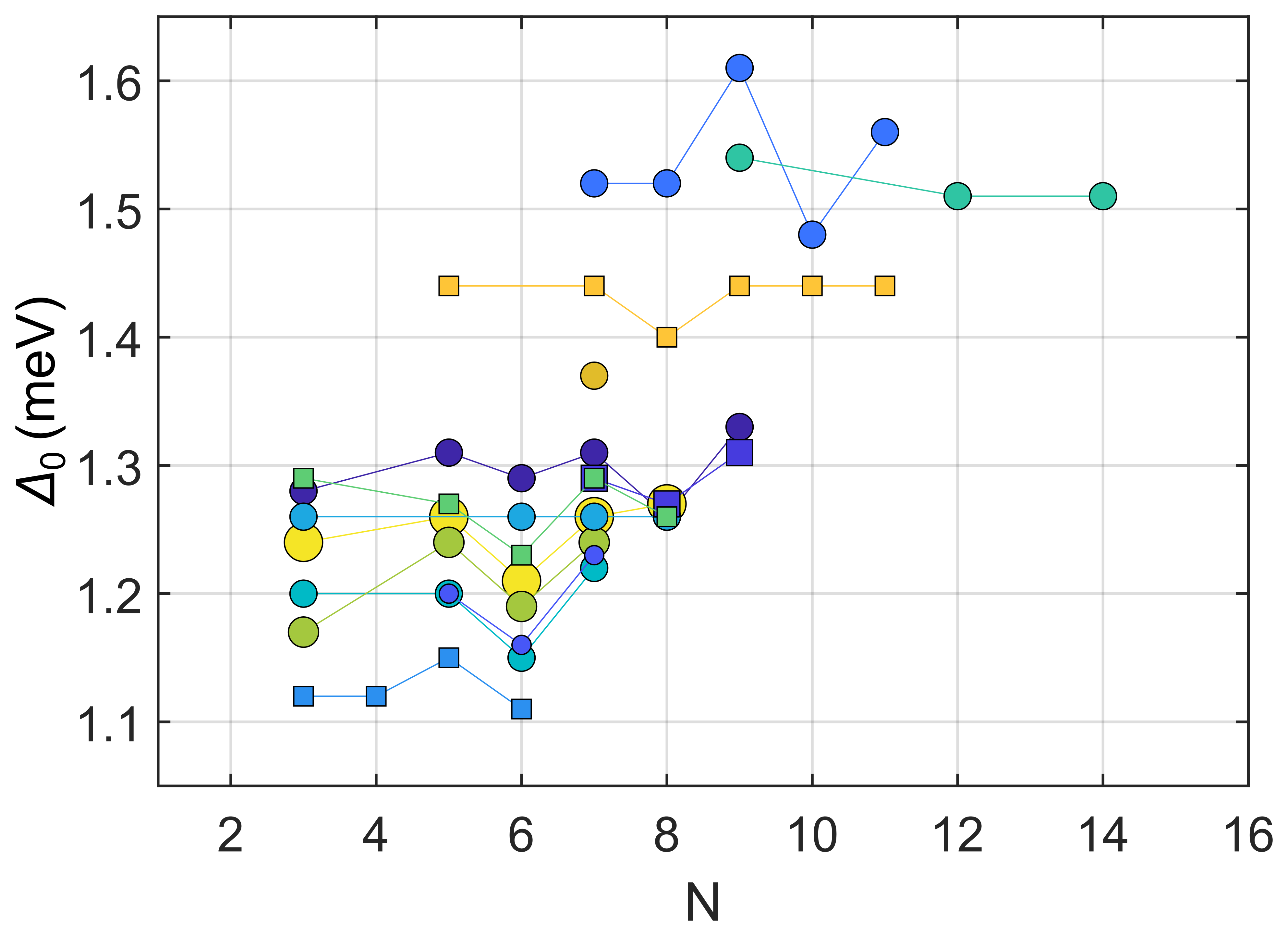


Supplementary Fig. 13. Thickness-dependence of the superconducting gap. The size of the anisotropic gap extracted from numerical fits is plotted as a function of layer thickness for different growths. Filled squares (circles) correspond to data measured at *T* = 30 mK (*T* = 1.3 K). For clarity, all points within the same growth are connected with solid lines. The estimated error in the gap value from the fitting is smaller than the symbol size, including the data in Fig. 5d (*T* = 30 mK), which is a subset of Supplementary Fig. 13.


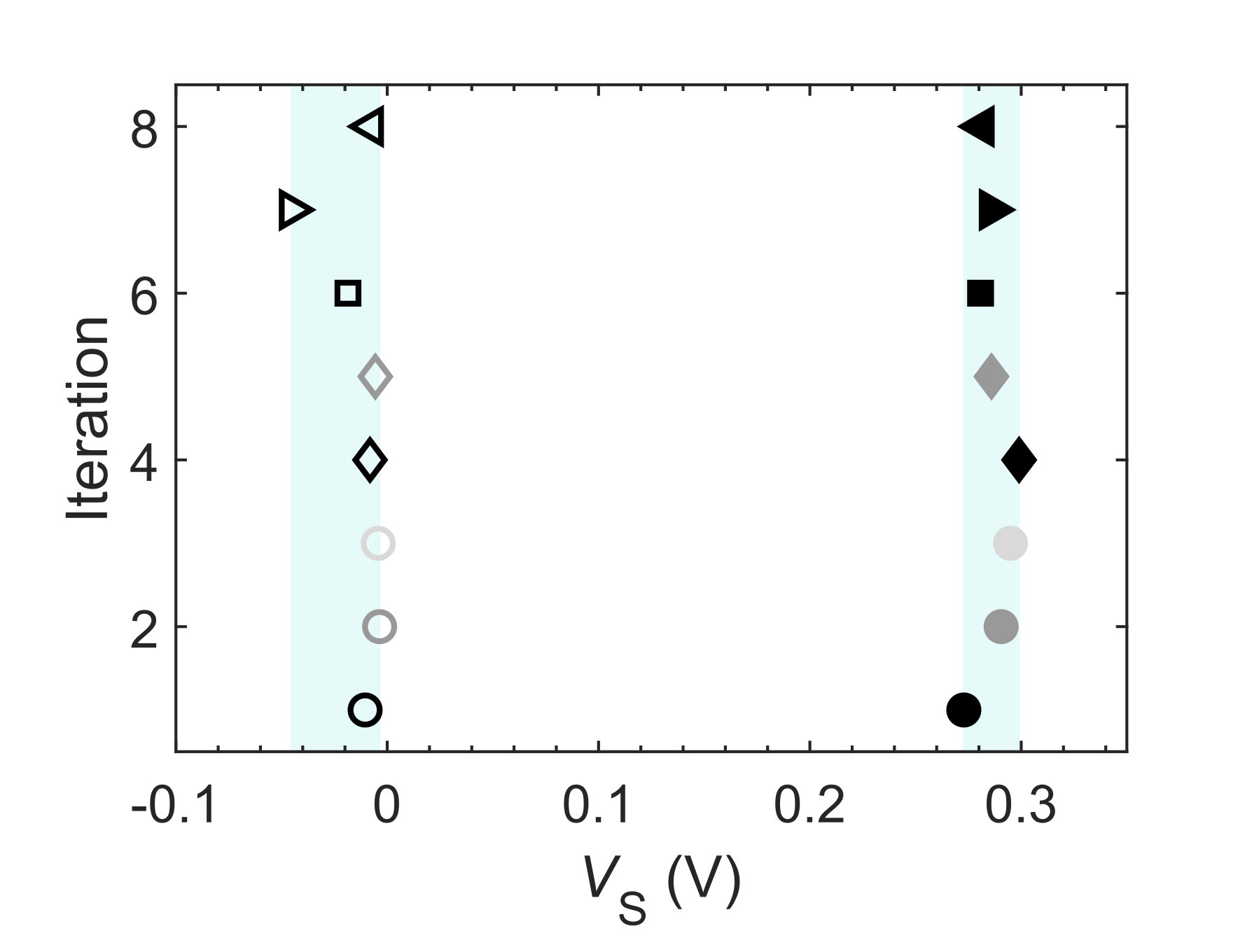


Supplementary Fig. 14. Bandgap measurement of pristine BP samples. The plot shows the conduction band edges (filled symbols) and valence band edges (empty symbols) for different BP samples determined from d*I*/d*V* spectra measured on the cleaved surface at *T* = 4.4 K ^4-6^. Multiple cleaves on the same BP crystal are shown with the same symbols. The light blue color in the background represents the variation in the position of the band edges. The estimated error in the band edges is smaller than the symbol sizes. (Stabilization parameters for d*I*/d*V* spectra: *V*_stab_ = -200 mV, *I*_stab_ = 20-200 pA, *V*_mod_ = 4-10 mV).

# Supplementary Table S1: Parameters used to fit the spectra in **Supplementary Fig.** **12**.

The table summarizes different parameters used for fitting spectra in Supplementary Fig. 12 as described in the methods section in the main manuscript.

| Spectrum | Measurement *T* (K) | *T*_eff_ (K) | $\Delta_{0}$ (meV) | $\zeta$ | $\epsilon$ | *p*_0_ | $\tau$ | $\chi$ | $\sigma$ |
| --- | --- | --- | --- | --- | --- | --- | --- | --- | --- |
| 1 | 30 m | 150 m | 1.11 | 0.005 | 10 | 2 | 0.1 | -1 | -5 |
| 2 | 30 m | 150 m | 1.29 | 0.009 | 10 | 2 | 0.2 | -0.5 | -4.5 |
| 3 | 30 m | 150 m | 1.29 | 0.015 | 10 | 1.5 | 0.15 | -0.9 | -1.5 |
| 4 | 30 m | 150 m | 1.44 | 0.01 | 10 | 2 | 0.15 | -0.9 | -3.8 |
| 5 | 1.3 | 1.3 | 1.22 | 0.035 | 10 | 1.5 | 0.1 | -0.6 | -3.1 |
| 6 | 1.3 | 1.3 | 1.26 | 0.017 | 10 | 1.5 | 0.1 | -0.6 | -2.3 |
| 7 | 1.3 | 1.3 | 1.31 | 0.002 | 10 | 1.5 | 0.1 | -0.6 | -1.4 |
| 8 | 1.3 | 1.3 | 1.52 | 0.025 | 10 | 1.5 | 0.1 | -1.2 | -1.5 |

# Supplementary references

1. Kamber U*, et al.* Moiré-induced electronic structure modifications in monolayer V_2_S_3_ on Au(111). *Physical Review B* **103**, 115414 (2021).

2. Zeller P, Günther S. What are the possible moiré patterns of graphene on hexagonally packed surfaces? Universal solution for hexagonal coincidence lattices, derived by a geometric construction. *New Journal of Physics* **16**, 083028 (2014).

3. Popescu V, Zunger A. Extracting ***E*** versus ***k*** effective band structure from supercell calculations on alloys and impurities. *Physical Review B* **85**, 085201 (2012).

4. Kiraly B*, et al.* Anisotropic Two-Dimensional Screening at the Surface of Black Phosphorus. *Physical Review Letters* **123**, 216403 (2019).

5. Knol EJ, Kiraly B, Rudenko AN, van Weerdenburg WMJ, Katsnelson MI, Khajetoorians AA. Gating orbital memory with an atomic donor. *arXiv e-prints*, arXiv:2107.07143 (2021).

6. Kiraly B, Hauptmann N, Rudenko AN, Katsnelson MI, Khajetoorians AA. Probing Single Vacancies in Black Phosphorus at the Atomic Level. *Nano Letters* **17**, 3607-3612 (2017).
